# Linking Heart Function to Prognosis: The Role of a Novel Echocardiographic Index and NT-proBNP in Acute Heart Failure

**DOI:** 10.3390/medicina61081412

**Published:** 2025-08-04

**Authors:** Dan-Cristian Popescu, Mara Ciobanu, Diana Țînț, Alexandru-Cristian Nechita

**Affiliations:** 1 Department of Cardiology, Clinical Emergency Hospital “Sfântul Pantelimon”, 021652 Bucharest, Romania; popescu_dan_95@yahoo.com (D.-C.P.); mara2228@gmail.com (M.C.); cardionechita@yahoo.com (A.-C.N.); 2 Faculty of Medicine, “Carol Davila” University of Medicine and Pharmacy, 020021 Bucharest, Romania; 3 Faculty of Medicine, “Transilvania” University, 500036 Brașov, Romania; 4 Department of Cardiology, ICCO Clinics, 50059 Brașov, Romania

**Keywords:** acute heart failure, echocardiography, right ventricular function, TAPSE, VTI LVOT, NT-proBNP, prognosis, mortality

## Abstract

*Background and Objectives:* Risk stratification in acute heart failure (AHF) remains challenging, particularly in settings where biomarker availability is limited. Echocardiography offers valuable hemodynamic insights, but no single parameter fully captures the complexity of biventricular dysfunction and pressure overload. This study aimed to evaluate a novel echocardiographic index (**ViRTUE Index**–*VTI-RVRA-TAPSE Unified Evaluation*) integrating a peak systolic gradient between the right ventricle and right atrium (RV-RA gradient), tricuspid annular plane systolic excursion (TAPSE), the velocity–time integral in the left ventricular outflow tract (VTI LVOT), NT-proBNP (N-terminal pro–B-type Natriuretic Peptide) levels, and in-hospital mortality among patients with AHF. *Materials and Methods:* We retrospectively analyzed 123 patients admitted with AHF. Echocardiographic evaluation at admission included TAPSE, VTI LVOT, and the RV-RA gradient. An index was calculated as $\frac{RV-RA\;gradient\;}{TAPSE\;x\;VTI\;LVOT}$. NT-proBNP levels and in-hospital outcomes were recorded. Statistical analysis included correlation, logistic regression, and ROC curve evaluation. *Results:* The proposed index showed a significant positive correlation with NT-proBNP values (r = 0.543, *p* < 0.0001) and good discriminative ability for elevated NT-proBNP (AUC = 0.79). It also correlated with in-hospital mortality (r = 0.193, *p* = 0.032) and showed moderate prognostic performance (AUC = 0.68). Higher index values were associated with greater mortality risk. *Conclusions:* This novel index, based on standard echocardiographic measurements, reflects both systolic dysfunction and pressure overload in AHF. Its correlation with NT-proBNP and in-hospital mortality highlights its potential as a practical, accessible bedside tool for early risk stratification, particularly when biomarker testing is unavailable or delayed.

## 1. Introduction

Acute heart failure (AHF) is a critical condition in cardiovascular medicine, commonly associated with frequent rehospitalizations and high early mortality rates [1,2]. Timely identification of risk is essential for improving clinical outcomes and informed treatment decisions in AHF patients [3,4,5]. Transthoracic echocardiography is among the most valuable diagnostic modalities, enabling fast, non-invasive assessment of cardiac anatomy and performance.

Various echocardiographic parameters have been investigated for their prognostic relevance in AHF [6], especially those reflecting biventricular systolic performance [7]. Although left ventricular ejection fraction (LVEF) remains a widely adopted marker, its diagnostic and prognostic limitations are well known, particularly in individuals with preserved systolic function. In recent years, global longitudinal strain (GLS) has become increasingly recognized as a more sensitive and consistent index, providing superior prognostic insights in acute settings [8,9].

Beyond LVEF and GLS, the left ventricular outflow tract velocity–time integral (VTI LVOT) has been suggested as a reliable estimate of stroke volume and cardiac output, with documented prognostic significance in both acute and chronic heart failure scenarios [10,11]. Evidence also indicates that VTI LVOT values ≤ 17 cm are associated with increased risk of cardiovascular death in patients with systolic dysfunction and mitral regurgitation [12].

Right ventricular (RV) function, although historically underrecognized, is now acknowledged as a key prognostic component in AHF [13]. Commonly assessed parameters include tricuspid annular plane systolic excursion (TAPSE), fractional area change (FAC), tissue Doppler-derived S’ velocity, and RV free-wall strain [14]. TAPSE is widely adopted in clinical practice because it is easy to obtain, reliable, and strongly linked with survival prognosis [15].

Additionally, elevated pulmonary pressures estimated via the RV-to-right-atrial (RV-RA) gradient or pulmonary artery systolic pressure (PASP) represent a significant hemodynamic burden on the right heart and are linked to unfavorable outcomes [16]. The TAPSE/PASP ratio serves as a noninvasive indicator of RV–pulmonary artery coupling and has been recognized as a strong prognostic marker in heart failure [17]. Studies show that TAPSE/PASP values below 0.4 mm/mmHg are strongly associated with increased mortality in AHF [18].

Given the complex pathophysiology of AHF, multiple studies have attempted to create composite echocardiographic indexes that reflect both functional and pressure-related derangements, aiming for a more integrated risk assessment [19].

In this context, our study proposes a new echocardiographic index (**ViRTUE Index**–*VTI-RVRA-TAPSE Unified Evaluation*) derived from three routine variables: the RV-RA gradient, TAPSE, and the VTI LVOT to quantify right and left ventricular systolic dysfunction and right-sided pressure overload. We hypothesize that this index correlates with NT-proBNP levels and in-hospital mortality, potentially serving as a practical alternative for early risk assessment in patients for whom advanced data are delayed or unavailable.

## 2. Materials and Methods

### 2.1. Study Design and Patient Population

This was a retrospective observational study conducted in the Cardiology Department of the Emergency Clinical Hospital “Sf. Pantelimon” in Bucharest. A total of 123 adult patients consecutively admitted for acute heart failure (AHF) between January and December 2024 were enrolled. The study was conducted in accordance with the ethical principles of the Declaration of Helsinki and was approved by the institutional ethics committee.

Inclusion criteria consisted of a confirmed diagnosis of AHF in accordance with the 2021 European Society of Cardiology guidelines [20], with a full clinical, laboratory, and echocardiographic evaluation performed on admission.

Exclusion criteria included acute coronary syndrome, myocarditis, Takotsubo cardiomyopathy, cardiac amyloidosis, hypertrophic cardiomyopathy, or incomplete data related to NT-proBNP, TAPSE, the VTI LVOT, or the RV-RA gradient. Patients diagnosed with Takotsubo cardiomyopathy, myocarditis, or hypertrophic cardiomyopathy were excluded from the study due to their distinct pathophysiological mechanisms and echocardiographic profiles, which differ significantly from typical acute heart failure syndromes. These conditions often present with transient, non-ischemic myocardial dysfunction (as in Takotsubo), inflammatory myocardial injury (as in myocarditis), or structural abnormalities unrelated to pressure or volume overload (as in hypertrophic cardiomyopathy). Including such cases could have introduced heterogeneity in echocardiographic parameters and confounded the correlation between the proposed index and NT-proBNP or in-hospital mortality. To ensure the internal validity and pathophysiological consistency of the cohort, these entities were systematically excluded.

### 2.2. Data Collection

Patients underwent clinical evaluation and laboratory testing at presentation in the emergency department, following the institutional protocol. Data collected included cardiovascular risk factors (e.g., smoking status, obesity, hypertension, diabetes, dyslipidemia), relevant medical history (prior myocardial infarction, cardiomyopathies, valvular diseases, arrhythmias, anemia, pulmonary disease), as well as findings from physical examination (blood pressure, heart rate, oxygen saturation, and signs of congestion such as dyspnea, edema, and pulmonary rales).

Laboratory tests comprised complete blood count, serum sodium and potassium, creatinine, urea, high-sensitivity troponin, NT-proBNP, C-reactive protein, Aspartate Aminotransferase (AST) and Alanine Aminotransferase (ALT), total and direct bilirubin, the international normalized ratio (INR), and serum albumin.

### 2.3. Echocardiographic Assessment

Transthoracic echocardiography was performed using a General Electric Vivid E95 ultrasound machine. All measurements were obtained from three consecutive cardiac cycles in sinus rhythm and interpreted by an experienced cardiologist blinded to patient outcomes.

The following parameters were measured:

TAPSE (tricuspid annular plane systolic excursion) was acquired in M-mode from the apical four-chamber view, reflecting the longitudinal systolic function of the right ventricle. Values ≥ 17 mm are generally considered normal.

The VTI LVOT (velocity–time integral of the left ventricular outflow tract) was assessed via pulsed-wave Doppler in the apical five-chamber view. This parameter reflects stroke volume and cardiac output. Normal values typically exceed 18 cm.

The RV-RA gradient was estimated using the simplified Bernoulli equation from the peak velocity of the tricuspid regurgitation jet. This value correlates with pulmonary artery systolic pressure and is usually considered normal below 35 mmHg.

Left ventricular ejection fraction (LVEF) was calculated using the biplane Simpson method. Additionally, valvular heart disease (especially mitral and aortic stenosis or regurgitation) was qualitatively assessed and graded based on current echocardiographic guidelines as mild, moderate, or severe [14].

### 2.4. Definition of the Echocardiographic Index

A novel echocardiographic index was calculated using the following formula:

$$INDEX=\frac{RV-RA\;gradient\;}{TAPSE\;x\;VTI\;LVOT}$$

where the RV-RA gradient is the peak systolic gradient between the right ventricle and right atrium (mmHg), TAPSE is the tricuspid annular plane systolic excursion (mm), and the VTI LVOT is the velocity–time integral in the left ventricular outflow tract (cm).

This index integrates parameters reflecting right ventricular systolic pressure, right ventricular longitudinal function, and left ventricular systolic output. It was calculated for each patient with complete echocardiographic data available.

### 2.5. Statistical Analysis

Continuous variables were reported as mean ± standard deviation (SD) or as median and interquartile range (IQR), depending on their distribution assessed with the Shapiro–Wilk test. Variables with a normal distribution were compared using the independent samples *t*-test, while non-normally distributed variables, including NT-proBNP (*p* < 0.001), were analyzed using the Mann–Whitney U test. Categorical variables were summarized as counts and percentages and compared using the chi-square test.

Correlation between the index and NT-proBNP levels was assessed using Spearman’s rank correlation. Binary logistic regression was applied to evaluate the association between individual echocardiographic parameters and dichotomized NT-proBNP levels, using the median value as the threshold. The diagnostic performance of the index was evaluated using ROC (receiver operating characteristic) curves. The area under the curve (AUC) was calculated to determine discriminatory ability. Optimal cutoff values were identified via Youden’s index. A *p*-value < 0.05 was considered statistically significant. Analyses were performed using Statistical Package for the Social Sciences (SPSS) version 26.0 and Jeffrey’s Amazing Statistics Program (JASP) version 0.19. AI-assisted tools (ChatGPT, OpenAI, 2025) were employed to support the generation and formatting of visual data representations (e.g., graphs).

## 3. Results

### 3.1. Baseline Characteristics

Between January and December 2024, a total of 123 patients admitted for acute heart failure were enrolled in the study. The mean age of the population was 71.3 ± 14.0 years, and 50.4% were male. Regarding cardiovascular risk factors, 47.15% of patients had a history of hypertension, 32.52% were diabetic, and 42.28% were diagnosed with dyslipidemia. Active smokers represented 22.76% of the cohort. The prevalence of ischemic etiology was 39.8%, while 24.4% had documented valvular heart disease. Atrial fibrillation was present in 34.1% of patients. Although NT-proBNP values are reported in Table 1 as mean ± standard deviation for consistency, this variable had a non-normal distribution (Shapiro–Wilk *p* < 0.001). Therefore, statistical comparison between survivors and non-survivors was performed using the Mann–Whitney U test, which revealed significantly higher levels in non-survivors (median [IQR]: 15,150 [9853.5–26,610.8] pg/mL) than in survivors (4173 [1569.5–8457.0] pg/mL), with *p* < 0.001. Demographic, clinical, laboratory, and echocardiographic characteristics of survivors and non-survivors are detailed in Table 1.

### 3.2. Relationship Between the Echocardiographic Index and NT-proBNP

All three echocardiographic parameters used in the index—the RV-RA gradient, TAPSE, and the VTI LVOT—were independently associated with elevated NT-proBNP levels in univariate logistic regression analysis (Table 2). For this analysis, NT-proBNP values were dichotomized at the median threshold of 4173 pg/mL.

There was a statistically significant positive correlation between the proposed echocardiographic index and NT-proBNP concentrations (Spearman r = 0.543, *p* < 0.0001), indicating a moderate association between higher index values and elevated levels of this biomarker. Although statistically meaningful, the visual trend observed in the scatter plot was modest, reflecting expected biological variability in acute heart failure presentations.

### 3.3. Predictive Accuracy of the Index for Elevated NT-proBNP

**Figure 1:**
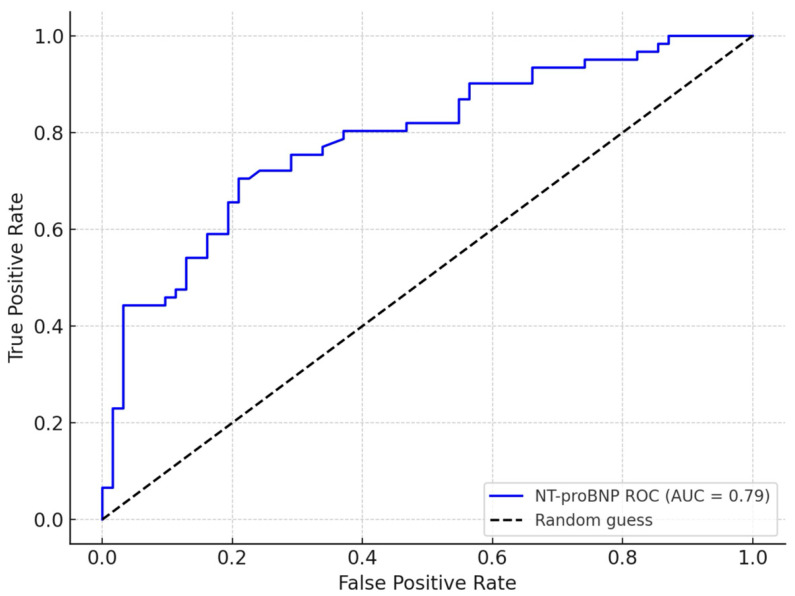
ROC analysis of the echocardiographic index for detecting high NT-proBNP (≥4173 pg/mL). NT-proBNP was dichotomized at the median. AUC: area under the curve.

The ROC curve assessing the index’s ability to predict elevated NT-proBNP yielded an area under the curve (AUC) of 0.79, suggesting good discriminative power. The optimal cutoff value of the index was determined to be 0.138, as per Youden’s index, with a sensitivity of 76% and specificity of 73% for identifying elevated NT-proBNP (Figure 1).

**Figure 2:**
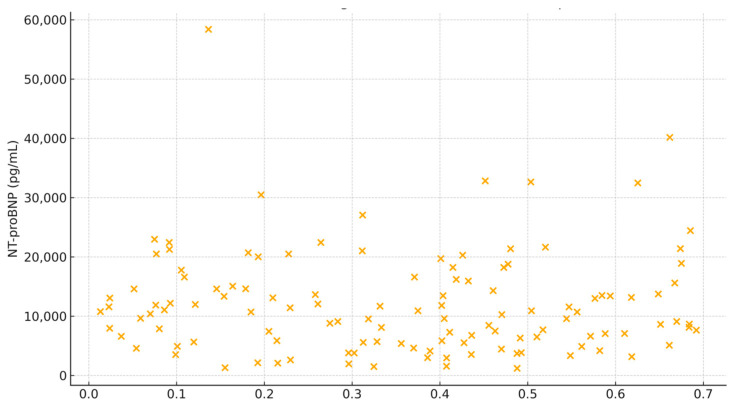
Scatter plot showing the relationship between the echocardiographic index and NTproBNP levels.

The scatter plot (Figure 2) suggests a moderate upward trend in NT-proBNP levels with increasing values in the index, as confirmed by logarithmic analysis (Spearman r = 0.543, *p* < 0.0001). This finding has several clinical and physiological implications. Firstly, it illustrates the inverse relationship between ventricular function and NT-proBNP: TAPSE (a marker of right ventricular function) and the VTI LVOT (reflecting left ventricular systolic output) decrease as NT-proBNP increases, indicating impaired function. Secondly, the RV-RA gradient is directly proportional to NT-proBNP, consistent with elevated pulmonary pressures and right ventricular strain. Overall, the index integrates these components to capture the combined effect of pressure overload and systolic dysfunction, both of which contribute to elevated NT-proBNP levels in acute heart failure.

### 3.4. Index Distribution by NT-proBNP Category

The proposed index integrates pulmonary pressures (RV-RA the gradient) and biventricular systolic function (TAPSE and the VTI LVOT). Higher index values reflect an imbalance between increased pressures and ventricular dysfunction, explaining the positive correlation with NT-proBNP and making it an effective integrative predictor of heart failure severity.

**Figure 3:**
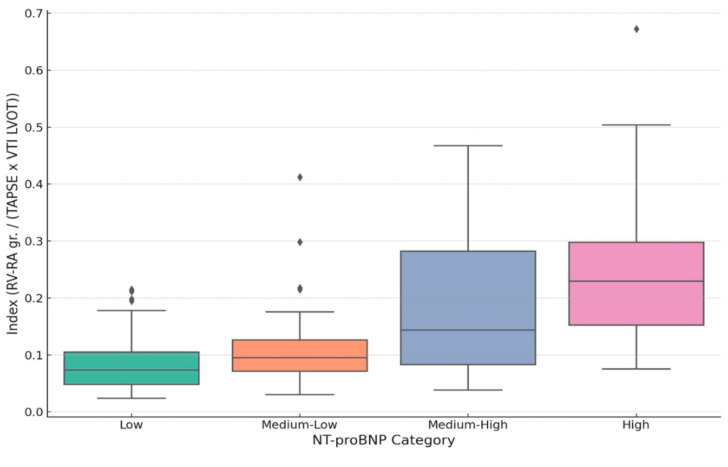
Distribution of the echocardiographic index values across NTproBNP categories.

**Figure 4:**
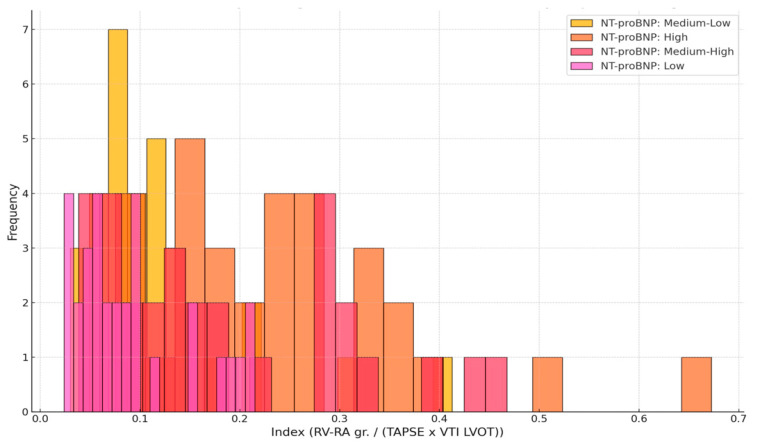
Histogram of the proposed index across NTproBNP categories in patients with acute heart failure.

Histograms and distribution graphs demonstrated a progressive shift toward higher index values across increasing NT-proBNP categories (low, intermediate, and high). Patients in the lowest NT-proBNP group generally exhibited index values < 0.2, while those in a higher NT-proBNP group more frequently had index values exceeding 0.3 (Figure 3 and Figure 4).

### 3.5. Correlation with In-Hospital Mortality

**Figure 5:**
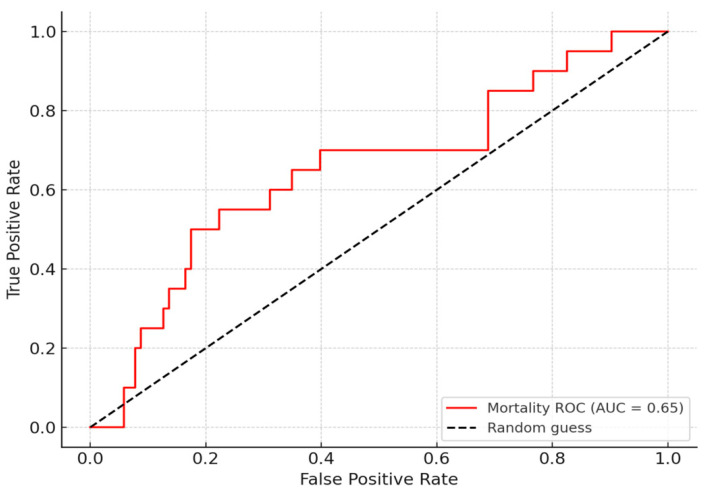
ROC curve assessing the predictive value of the echocardiographic index for in-hospital mortality.

The proposed index showed a statistically significant, though weak, correlation with in-hospital mortality (Spearman r = 0.193, *p* = 0.032). The ROC analysis yielded an AUC of 0.65, reflecting only modest discriminatory ability. Although this value does not support the index as a standalone predictor, it may still offer complementary prognostic information when integrated into broader risk stratification models in acute heart failure (Figure 5).

While it is not a perfect predictor, the index provides valuable clinical insights by integrating echocardiographic parameters that are easily obtained in routine clinical practice.

**Figure 6:**
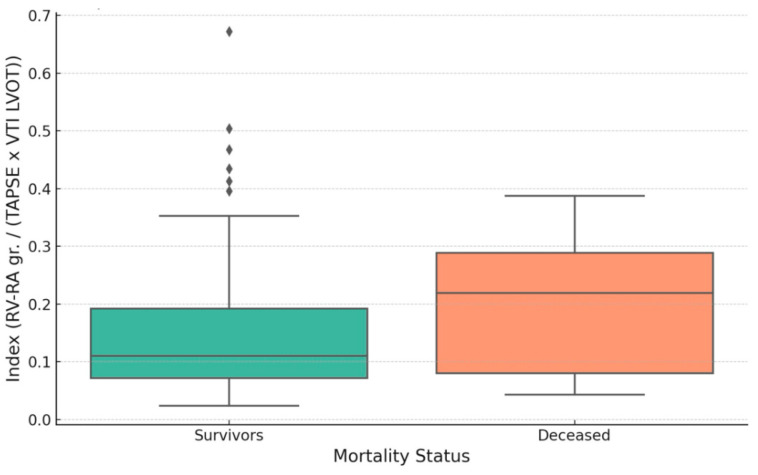
Bar chart comparing in-hospital mortality rates above and below the index cutoff of 0.209 (*p* = 0.023).

Using Youden’s index, the optimal cutoff value for predicting in-hospital death was calculated at 0.209. Patients with values exceeding this threshold demonstrated a higher risk of in-hospital mortality (*p* = 0.023; Figure 6).

### 3.6. Multivariable Analysis of Mortality Predictors

In a multivariable logistic regression model including age, renal function (eGFR), left ventricular ejection fraction (LVEF), and NT-proBNP, the index was not independently associated with in-hospital mortality (OR = 1.12, *p* = 0.962). NT-proBNP remained a strong predictor (OR = 1.55, *p* < 0.001), suggesting that neurohormonal activation may capture additional prognostic information beyond hemodynamic parameters alone.

## 4. Discussion

### 4.1. Pathophysiological Interpretation

NT-proBNP is widely recognized as a biomarker of myocardial strain, fluid overload, and increased intracardiac pressures [21,22]. In this study, the newly developed echocardiographic index exhibited a strong association with NT-proBNP concentrations, reflecting the interplay between mechanical dysfunction and neurohormonal activation.

The underlying pathophysiology supports this relationship: a diminished TAPSE indicates impaired right ventricular longitudinal contractility; a low VTI LVOT suggests a reduced stroke volume and an elevated RV-RA gradient reflects pulmonary hypertension and right ventricular increased afterload [15,16]. These elements capture both systolic dysfunction and hemodynamic congestion.

Recent research further supports the prognostic value of these metrics, showing that TAPSE/PASP ratios below 0.4 mm/mmHg are associated with higher in-hospital mortality [18], and that a VTI LVOT ≤ 17 cm correlates with increased cardiovascular risk [12]. These findings validate the rationale for integrating such parameters into a composite index.

Unlike NT-proBNP, which can be influenced by extracardiac variables such as renal impairment and obesity [23], the index provides a direct echocardiographic assessment of hemodynamic function.

Moreover, a 2024 study demonstrated that RV global longitudinal strain corrected by PASP (RV-GLS/PASP) outperformed TAPSE/PASP in the prognostic stratification of AHF patients [24].

### 4.2. Clinical Relevance

All components of the index are part of standard echocardiographic evaluation and require no additional software or imaging technology. This makes the index particularly useful in acute care settings or in centers with limited access to biomarker assays.

The significant correlation with NT-proBNP and its association with mortality risk suggest that the index can aid clinicians in quickly identifying patients who may benefit from the early escalation of therapy or closer monitoring.

### 4.3. Comparison with the Existing Literature

The results are in line with previous studies highlighting the predictive value of TAPSE and the VTI LVOT in heart failure [10,16]. Furthermore, meta-analytic evidence confirms the robustness of the TAPSE/PASP ratio across various heart failure phenotypes, recommending a cutoff of approximately 0.36 mm/mmHg for mortality prediction [25].

Biomarker-imaging correlations have also been studied, with NT-proBNP levels shown to reflect the degree of both left and right ventricular dysfunction seen on echocardiography [26]. These studies underscore the complementary nature of imaging and laboratory parameters in risk stratification.

In our multivariable logistic regression analysis, the proposed index was not independently associated with in-hospital mortality after adjustment for key clinical and echocardiographic variables, including age, renal function, ejection fraction, and NT-proBNP. Notably, NT-proBNP remained a strong predictor of adverse outcomes. These results are consistent with the modest univariable discriminatory performance observed (AUC = 0.65) and support the interpretation that the index may serve as a complementary, rather than standalone, prognostic marker (Table 3).

While the index integrates components from both ventricles and the pulmonary circulation, further research is needed to directly compare its prognostic value with established ratios such as TAPSE/PASP or RV-GLS/PASP. Such analyses would clarify whether the index provides incremental or superior risk stratification.

Although we acknowledge the value of formal statistical comparisons such as the AUC and net reclassification improvement (NRI), these were not performed in the current study due to the limited number of in-hospital mortality events and the absence of complete PASP data for TAPSE/PASP calculation across all patients. While PASP could potentially be estimated in some patients to allow for TAPSE/PASP calculation, this would require assumptions regarding right atrial pressure and consistent TR signal quality, which were not uniformly available in our retrospective dataset. We consider these methods a priority for future validation efforts using prospectively collected data.

### 4.4. Strengths and Limitations

The main strengths of this research include the use of a real-world cohort of consecutive AHF patients, the application of commonly available echocardiographic parameters, and the robust statistical methodology including correlation and ROC analysis.

However, several limitations must be acknowledged: the study was conducted in a single center, using a retrospective design, and included a relatively limited sample size. Although left ventricular ejection fraction (LVEF) was included as a covariate in multivariable models, we were not able to perform formal stratified analyses by heart failure phenotypes (e.g., HFrEF, HFpEF) due to the low number of events per subgroup.

### 4.5. Future Perspectives

Future prospective and multicenter trials are needed to confirm the prognostic performance of this index and its reproducibility in different patient populations. Comparative studies with established indices such as TAPSE/PASP, GLS, and NT-proBNP + troponin combinations may further clarify its incremental value. Moreover, evaluating whether the index can guide therapeutic strategies or predict long-term events such as 30-day readmission or mortality could enhance its clinical applicability.

Future studies should aim to validate the prognostic performance of the index in larger, multicenter cohorts and across various clinical settings. In particular, evaluating the index within heart failure subtypes (HFrEF, HFmrEF, HFpEF) would help determine whether its predictive value is consistent across different phenotypes. Additionally, prospective studies could explore its dynamic behavior under changing preload and afterload conditions, and its potential incremental value over established prognostic tools such as TAPSE/PASP or RV-GLS/PASP.

Considering the emerging recognition of right ventricular dysfunction as a key determinant of outcomes in acute heart failure, future research should place greater emphasis on assessing and integrating right heart performance into prognostic models.

## 5. Conclusions

This study evaluated a novel echocardiographic index offering a comprehensive assessment by integrating parameters from both ventricles and the pulmonary circulation, thereby reflecting the complex pathophysiology of acute heart failure. Its components are routinely obtainable during emergency echocardiography and allow for immediate bedside calculation without the need for advanced software. This simplicity and accessibility make the index particularly useful for urgent decision-making and triage in acute care settings.

The index demonstrated a significant correlation with NT-proBNP levels (r = 0.543, *p* < 0.0001), indicating that it effectively captures the degree of neurohormonal activation and hemodynamic burden. Its diagnostic performance in identifying patients with an elevated NT-proBNP was good (AUC = 0.79), supporting its potential use as a surrogate marker when laboratory tests are delayed.

Additionally, the index showed a moderate association with in-hospital mortality (AUC = 0.68), suggesting that it reflects some clinically relevant aspects of biventricular dysfunction and pressure overload. Although this level of discrimination is limited, the index may still assist clinicians in early risk triage, especially when used alongside other prognostic tools.

## Figures and Tables

**Table 1:** Baseline characteristics of the study population according to in-hospital survival status.

Variable	Total (N = 123)	Survivors (N = 103)	Non-Survivors (N = 20)	*p*-Value
Age (years)	71.34 ± 13.98	70.3 ± 14.34	76.4 ± 10.85	0.039
Male sex, %	50.4	50.4	50.0	0.968
Smoking, %	22.76	25.24	10.0	0.137
Hypertension, %	86.99	88.34	80.0	0.310
Dyslipidemia, %	91.86	95.14	75.0	0.003
Obesity, %	39.83	35.92	60.0	0.044
Diabetes mellitus, %	39.83	38.83	45.0	0.606
Systolic BP (mmHg)	142.59 ± 29.54	144.33 ± 27.43	133.6 ± 38.23	0.244
Diastolic BP (mmHg)	85.83 ± 16.68	87.84 ± 15.75	75.45 ± 17.91	0.002
Heart rate (bpm)	100.98 ± 28.12	101.5 ± 29.18	98.3 ± 22.32	0.644
NT-proBNP (pg/mL)	9228.3 ± 9736.4	7569.6 ± 8565.9	17,770.8 ± 11,093.4	0.001
TAPSE (mm)	18.32 ± 5.16	18.53 ± 5.09	17.25 ± 5.49	0.309
VTI LVOT (cm)	15.5 ± 5.22	15.97 ± 5.30	13.07 ± 4.06	0.022
RV-RA gradient (mmHg)	33.64 ± 12.14	33.09 ± 12.05	37.0 ± 12.44	0.190
LVEF (%)	41.21 ± 16.78	41.15 ± 16.58	41.5 ± 18.23	0.934
Valvular heart disease, %	73.17	71.84	80.0	0.529

BP, blood pressure; bpm, beats per minute; NT-proBNP, N-terminal prohormone of brain natriuretic peptide; TAPSE, tricuspid annular plane systolic excursion; VTI LVOT, velocity–time integral in the left ventricular outflow tract; RV-RA gradient, peak systolic gradient between the right ventricle and right atrium; LVEF, left ventricle ejection fraction; NT-proBNP is reported as mean ± SD for consistency, although its distribution was non-normal (Shapiro–Wilk *p* < 0.001).

**Table 2:** Logistic regression results for evaluating the correlation between echocardiographic parameters and NT-proBNP levels in patients with acute heart failure.

Variable	Odds Ratio	*p*-Value	CI 95%
VTI LVOT	0.86	0.002	0.775 to 0.943
RV-RA gradient	1.04	0.037	1.002 to 1.080
TAPSE	0.90	0.034	0.812 to 0.991

VTI LVOT, velocity–time integral in the left ventricular outflow tract; RV-RA gradient, peak systolic gradient between the right ventricle and right atrium; TAPSE, tricuspid annular plane systolic excursion.

**Table 3:** Multivariable logistic regression analysis for in-hospital mortality, including the proposed echocardiographic index, age, renal function, LVEF, and log-transformed NT-proBNP.

	OR	OR 95% CI Lower	OR 95% CI Upper	*p*-Value
Index (GRVDAD/TAPSE x IVTLVOT)	1.121555	0.010058	125.0652	0.961959
Age	1.019246	0.968348	1.072819	0.465779
eGFR at Admission	1.010532	0.981655	1.040259	0.478765
LVEF	25.99523	0.557571	1211.957	0.09652
log(NT-proBNP)	4.719458	1.989161	11.19732	0.000431

## Data Availability

The data presented in this study are available on request from the corresponding author. The data are not publicly available due to patient privacy and confidentiality restrictions.

## Abbreviations

AHF	Acute Heart Failure
ALT	Alanine Aminotransferase
AST	Aspartate Aminotransferase
AUC	Area Under the Curve
BP	Blood Pressure
EF	Ejection Fraction
FAC	Fractional Area Change
GLS	Global Longitudinal Strain
HF	Heart Failure
INR	International Normalized Ratio
IQR	Interquartile Range
IVT	Time-Velocity Integral
JASP	Jeffrey’s Amazing Statistics Program
LV	Left Ventricle
LVEF	Left Ventricular Ejection Fraction
LVOT	Left Ventricular Outflow Tract
NT-proBNP	N-terminal pro–B-type Natriuretic Peptide
PASP	Pulmonary Artery Systolic Pressure
ROC	Receiver Operating Characteristic
RV	Right Ventricle
RV-GLS	Right Ventricular Global Longitudinal Strain
RV-RA	Right Ventricular–Right Atrial
RVGLS/PASP	Ratio of RVGLS to PASP
SD	Standard Deviation
SPSS	Statistical Package for the Social Sciences
TAPSE	Tricuspid Annular Plane Systolic Excursion
TAPSE/PASP	Ratio of TAPSE to PASP
VTI	Velocity–Time Integral
